# Disruption of the *M949_RS01915* gene changed the bacterial lipopolysaccharide pattern, pathogenicity and gene expression of *Riemerella anatipestifer*

**DOI:** 10.1186/s13567-017-0409-6

**Published:** 2017-02-06

**Authors:** Yafeng Dou, Xiaolan Wang, Guijing Yu, Shaohui Wang, Mingxing Tian, Jingjing Qi, Tao Li, Chan Ding, Shengqing Yu

**Affiliations:** Shanghai Veterinary Research Institute, Chinese Academy of Agricultural Sciences, Shanghai, 200241 China

## Abstract

*Riemerella anatipestifer* is an important pathogen that causes septicemia anserum exsudativa in ducks. Lipopolysaccharide (LPS) is considered to be a major virulence factor of *R. anatipestifer*. To identify genes involved in LPS biosynthesis, we screened a library of random Tn*4351* transposon mutants using a monoclonal antibody against *R. anatipestifer* serotype 1 LPS (anti-LPS MAb). A mutant strain RA1067 which lost the reactivity in an indirect ELISA was obtained. Southern blot and sequencing analyses indicated a single Tn*4351* was inserted at 116 bp in the *M949_RS01915* gene in the RA1067 chromosomal DNA. Silver staining and Western blot analyses indicated that the RA1067 LPS was defected compared to the wild-type strain CH3 LPS. The RA1067 displayed a significant decreased growth rate at the late stage of growth in TSB in comparison with CH3. In addition, RA1067 showed higher susceptibility to complement-dependent killing, more than 360-fold attenuated virulence based on the median lethal dose determination, increased bacterial adhesion and invasion capacities to Vero cells and significantly decreased blood bacterial loads in RA1067 infected ducks, when compared to the CH3. An animal experiment indicated that inactivated RA1067 cells was effective in cross-protecting of the ducks from challenging with *R. anatipestifer* strains WJ4 (serotype 1), Yb2 (serotype 2) and HXb2 (serotype 10), further confirming the alteration of the RA1067 antigenicity. Moreover, RNA-Seq analysis and real-time PCR verified two up-regulated and three down-regulated genes in RA1067. Our findings demonstrate that the *M949_RS01915* gene is associated to bacterial antigenicity, pathogenicity and gene regulation of *R. anatipestifer*.

## Introduction

*Riemerella anatipestifer* is a Gram-negative, non-motile, non-spore-forming, rod-shaped bacterium, which causes epizootic infectious disease in poultry, especially in ducks [1–3]. *R. anatipestifer* infected ducks were characterized by airsacculitis, pericarditis, perihepatitis, diarrhoea, ataxia, meningitis and depression of growth rate [4]. Until now, 21 serotypes of *R. anatipestifer* have been identified, there is poor cross-protection among them [5–7]. The occurrence of different serotypes has been reported in China, and serotypes 1, 2, and 10 have been responsible for most of the major outbreaks [8].

Up to date, several virulence-associated genes, including VapD, CAMP cohemolysis, outer membrane protein and TonB-dependent receptor tbdr1 have been identified in *R. anatipestifer* strains [3, 9, 10]. Recently, 49 novel virulence genes were identified from a transposon mutant library [11]. Lipopolysaccharide (LPS), the main component of the outer membrane of Gram-negative bacteria, is a potent stimulant of innate immune response [12]. The LPS is composed of three distinct components: lipid A, O-antigen and core oligosaccharide. The O-antigen consists of oligosaccharide repeating units (O units), which usually contain two to eight residues from a broad range of sugars, both common and rare, and their derivatives. The diversity of the O-antigen repeats is displayed in the types of sugar conformation, their arrangement, and the linkages within and between O-units [13, 14]. Consequently, the O-antigen repeats are the most variable constituent of the LPS molecule, imparting the antigenic specificity.

The O-antigen repeats are synthesized in the cytoplasm and then transported to the periplasmic face of the inner membrane. In *Escherichia coli* and *Salmonella enteric*, genes required for the biosynthesis of O-antigen repeats are located between *galF* and *gnd* genes [15]. In *R. anatipestifer*, however, three genes of the *AS87_04050*, *M949_1556* and *M949_1603* were identified to be involved in O-antigen biosynthesis [16–18]. In this study, we obtained one mutant strain RA1067 that lost the reactivity with anti-LPS MAb by screening a random Tn*4351* transposon insertion library using a monoclonal antibody against *R. anatipestifer* serotype 1 LPS (anti-LPS MAb). Sequence analysis showed that the *M949_RS01915* gene was inactivated in the RA1067. Furthermore, the bacterial antigenicity, pathogenicity and gene expression of the RA1067 were characterized.

## Materials and methods

### Ethics

One-day old Cherry Valley ducks were obtained from Zhuang Hang Duck Farm (Shanghai, China) and reared under controlled temperature (28–30 °C). The ducks were accommodated in cages with free access to food and water under the conditions of biological safety. Animal experiments were carried out in agreement with the Institutional Animal Care and Use Committee (IACUC) guidelines set by Shanghai Veterinary Research Institute, the Chinese Academy of Agricultural Sciences (CAAS). This animal study protocol (Shvri-po-0176) was approved by the IACUC of Shanghai Veterinary Research Institute, CAAS, China.

### Bacterial strains, plasmids and culture conditions

The bacterial strains and plasmids used in this study are listed in Table 1. *R. anatipestifer* CH3 is the serotype 1, wild-type (WT) strain used in this study, and the mutant strain RA1067 was obtained from this strain by Tn*4351* insertion. *R. anatipestifer* strains were grown on Tryptic Soy Agar (TSA, Difco, NJ, USA) or in Tryptic Soy Broth (TSB, Difco) at 37 °C with 5% CO_2_. *Escherichia coli* strains were grown at 37 °C on Luria–Bertani (LB) plates or in LB broth. Antibiotics were used at the given concentrations when required: ampicillin (100 μg/mL), chloramphenicol (30 μg/mL), erythromycin (0.5 μg/mL), kanamycin (50 μg/mL), streptomycin (50 μg/mL) and cefoxitin (5 μg/mL).

**Table 1:** **Strains, plasmids and primers used in this study**

Strains, plasmids or primers	Characteristics	Source or references
*Strains*		
CH3	*Riemerella anatipestifer* serotype 1 strain	[8]
RA1067	Tn4351 insertion at M949_RS01915 gene mutant of *Riemerella anatipestifer* CH3	This study
BW19851(pEP*4351*)	Plasmid pEP*4351* in BW19851, CmR	[8]
WJ4	*R. anatipestifer* wild-type strain, serotype 1	[8]
Yb2	*R. anatipestifer* wild-type strain, serotype 2	[8]
HXb2	*R. anatipestifer* wild-type strain, serotype 10	[8]
*Primers*		
16S rRNA F	5′-GAGCGGTAGAGTATCTTCGGATACT-3′	This study
16S rRNA R	5′-AATTCCTTTGAGTTTCAACCTTGCG-3′	This study
TN-1	5′-GGACCTACCTCATAG-3′	This study
IS4351	5′-TCAGAGTGAGAGAAAGGG-3′	This study
Tn4351-F	5′-TGGCACCTTTGTGGTTCTTAC-3′	This study
Tn4351-R	5′-GAGAGACAATGTCCCCCTTTC-3′	This study
Erm-F	5′-GCCCGAAATGTTCAAGTTGT-3′	This study
Erm-R	5′-CTTGACAACCACCCGACTTT-3′	This study
M949_ RS01915F	5′-AGTCTGCGTTGATCACCTTT-3′	This study
M949_ RS01915R	5′-AGCAAATACCAACAGAAGGGA-3′	This study
M949_ RS01920F	5′-AACCTCACTATCGGACCAGG-3′	This study
M949_ RS01920R	5′-GCCCGTTCCCAATTATTTGC-3′	This study
M949_ RS01910F	5′-GGAACTGGGATAGACGACCA-3′	This study
M949 _RS01910R	5′-AGAGCAGAGCGTTTACCCTT-3′	This study
RA ldh- F	5′-AGAGGAGCTTATCGGCATCA-3′	This study
RA ldh -R	5′-CTAGGGCTTCTGCCAATCTG-3′	This study
RA1067-F	5′-ATGAATTATTTTAAACTGCT-3′	This study
RA1067-R	5′-TTAGTCTAATTTCTGTATAT-3′	This study

### Indirect ELISA

The anti-LPS MAb 8A9 was used to screen the Tn*4351* insertion mutants for loss of reactivity by an indirect ELISA [18]. Briefly, 96-well ELISA plates were coated with the mutant strain suspended in carbonate buffered saline (CBS, pH 9.6) at 10^9^ CFU/well in 50 μL, and then heat-dried overnight in a drying oven at 55 °C. After being washed three times with phosphate-buffered saline (PBS) containing 0.05% Tween 20 (PBST), the plates were blocked for 2 h at 37 °C in PBS containing 5% skim milk, washed with PBST, and then incubated for 2 h with the anti-LPS MAb, followed by incubation for 1.5 h with a horseradish peroxidase (HRP)-conjugated anti-mouse IgG polyclonal antibody (Tiangen, Beijing, China). The reaction was visualized by addition of 3,3′,5,5′-tetramethyl benzidine (TMB) (Tiangen) and stopped using 2 M·H_2_SO_4_ solution. The resulting OD_450_ values were obtained using a plate reader (Synergy 2; BioTeck). The mutant with the OD_450_ value of <2.1 times of negative wells was selected for further analysis. All the mutants were screened in triplicate. The WT strain CH3 was used as a positive coating control.

### Identification of a mutant strain

Polymerase chain reaction (PCR) was performed to identify the WT strain CH3 and mutant strain RA1067 using primers 16S rRNA F/16S rRNA R, Erm-F/Erm-R and RA1067-F/RA1067-R (Table 1). Southern blot analysis was used to identify transposon Tn*4351* insertion in the mutant strain genome [19]. Briefly, genomic DNA of the mutant strain was extracted using TIANamp Bacteria DNA kit (Tiangen), digested by XbaΙ, separated by gel electrophoresis, and transferred to a nylon membrane. After being washed with saline sodium citrate, the membrane was immobilized for 2 h at 80 °C. A probe was prepared using a DIG DNA labeling and detection kit (Roche, Indianapolis, IN, USA). Southern blot hybridization was performed by standard method in accordance with the manufacturer’s instructions. The plasmid pEP4351 and genomic DNA of the WT strain CH3 were also subjected to hybridization analysis, which were used as the positive and negative controls respectively.

Inverse PCR was used to determine the transposon insertion site in the mutant strain [20]. Briefly, genomic DNA of the mutant strain was digested with *Hin*dIII and ligated to form a closed circle. The DNA adjacent to the insertion site was amplified using Tn*4351*-specific primers TN-1 and IS4351-F. DNA sequencing data were compared to a database using BLAST from the National Center for Biotechnology Information (NCBI) website [21].

### LPS extraction, silver staining and Western blot

LPS was extracted from the WT strain CH3 and mutant strain RA1067 according to the instructions of the manufacturer of the LPS extraction Kit (iNtRON Biotechnology, Boca Raton, FL, USA). Purified LPS was analyzed by sodium dodecyl sulfate polyacrylamide gel electrophoresis (SDS-PAGE). Gels were stained with silver to visualize the presence of LPS [22], and stained with coomassie blue to exclude the contamination of protein.

For Western blot analysis, the purified LPS were separated by SDS-PAGE and then transferred onto nitrocellulose membranes (Millipore, Billerica, MA, USA). The membranes were blocked overnight at 4 °C in PBS containing 5% skim milk, washed with PBST and then incubated for 2 h with anti-LPS MAb, followed by incubation for 1 h with an IRDYE680CW-conjugated donkey anti-mouse IgG polyclonal antibody (LI-COR Biosciences, Lincoln, NE, USA). The blots were visualized with an Odyssey two-color infrared imaging system (LI-COR Biosciences).

### Adhesion and invasion assays

Adhesion and invasion assays were performed with Vero cells (ATCC CCL-81) as described [3]. Briefly, Vero cells (10^5^/well) were seeded into 24-well tissue culture trays in Dulbecco’s modified Eagle medium (DMEM), containing 10% fetal bovine serum (FBS, Biowest, France). Vero cells were grown for 24 h at 37 °C in a humidified incubator with 5% CO_2_ atmosphere, rinsed three times with sterile PBS and infected with approximately 10^7^ CFU of each strain, respectively. The infected cells were then incubated at 37 °C with 5% CO_2_ for 1.5 h, rinsed three times with sterile PBS and lysed with 0.1% trypsin (100 μL/well). The number of cell-adherent bacteria was determined after tenfold dilution and spreading onto TSA plates. For the invasion assay, extracellular bacteria were killed with 100 μg/mL gentamicin in DMEM medium by additional 1-h incubation after bacterial infection. After being washed three times with sterile PBS, the infected cells were lysed and the amount of intracellular bacteria was counted. All of the above assays were tested in triplicate and replicated three times.

### Bacterial growth curves and virulence determination

The growth curves of the WT strain CH3 and mutant strain RA1067 were measured as described previously [8]. The WT strain CH3 and mutant strain RA1067 were grown in TSB respectively at 37 °C for 8 h with shaking. The bacterial cultures were then inoculated into fresh TSB medium at a ratio of 1:100 (v/v) and incubated at 37 °C, with shaking at 200 rpm. Bacterial growth was measured by counting the number of bacterial CFU at 2 h intervals for 16 h.

To determine whether the *M949_RS01915* gene plays a role on virulence of *R. anatipestifer,* the bacterial median lethal doses (LD_50_) of the WT strain CH3 and mutant strain RA1067 were determined using 18-day-old Cherry Valley ducks as described [3]. The ducks were evenly divided into five groups (8 ducks/group), and injected intramuscularly with the bacterial strain at a dose of 10^6^, 10^7^, 10^8^, 10^9^, or 10^10^ CFU, respectively. Moribund ducks were euthanized humanely with an intravenous injection of sodium pentobarbital at a dose of 120 mg/kg and counted as dead. Dead ducks were subjected to *R. anatipestifer* identification. Ducks were monitored daily for clinical symptoms and death rate for a period of 7 days post-infection. LD_50_ value was calculated by the improved Karber’s method [23].

Seventeen-day-old Cherry Valley ducks were injected intramuscularly with 10^8^ CFU of the WT strain CH3 and mutant strain RA1067 to evaluate the bacterial survival in vivo. Blood samples were collected at 6, 12, 24 and 48 h after infection (six ducks per group at each time point), were diluted tenfold and plated on TSA plates for bacterial counting.

### Serum sensitivity assays

Bacterial susceptibility to normal duck sera was conducted as described [24], with modifications. Briefly, normal duck sera were collected from the 17-old-day healthy Cherry Valley ducks, pooled and filter-sterilized (0.22 um). Pooled duck sera were diluted to 12.5, 25, 50% (v/v) in pH 7.2 PBS. Each 10 μL of bacterial suspension containing 10^8^ CFU was added into 190 μL serial diluted duck sera, pooled duck sera without dilution, the heat-inactivated duck sera (56 °C, 30 min) and PBS, respectively. The reaction mixtures were incubated at 37 °C with 5% CO_2_ for 30 min, and then tenfold serial diluted and plated onto TSA plates. The plates were incubated at 37 °C with 5% CO_2_ for 28 h to count bacterial colonies.

### Cross-protection assessment

The inactivated vaccine was developed using the mutant strain RA1067 to evaluate the cross-protection. The inactivated CH3 vaccine was used as the WT strain control and developed as described [17]. A total of 72 ducks (7-day old) were divided into three groups of 24, received two immunizations at day 7 and 21 respectively with inactivated RA1067 vaccine (group 1, 5 × 10^8^ CFU bacterial cells in 0.3 mL vaccine), inactivated CH3 vaccine (group 2, 5 × 10^8^ CFU bacterial cells in 0.3 mL vaccine), as described [25]. The ducks in group 3 received two subcutaneous injections of saline in adjuvant as controls. At 2 weeks post-immunization, each eight ducks from each group were challenged with *R. anatipestifer* strain WJ4 (serotype 1), Yb2 (serotype 2) or HXb2 (serotype 10) by subcutaneous injection at a dose of 10 LD_50_ in 0.5 mL saline, respectively. Ducks were monitored and recorded daily for clinical symptoms and death until 7 days post-infection.

### Illumina sequencing for RNA-Seq and differential expression analysis

Total RNA quantification and quality were assessed by spectrophotometer, ribosome RNA were removed using Ribo-Zero™ Magnetic Gold Kit (epicenter, USA), then the protocol of TruSeq RNA Sample Prep Kit v2 (Illumina) to construct the libraries was followed. The complete libraries were sequenced for 100 cycles on Illumina HiSeq 2000 as described [26]. Image analysis and base calling were performed using Solexa pipeline Version 1.8 (Off-Line Base Caller software, Version 1.8) [27]. Cleaned reads were aligned to *R. anatipestifer* CH3 genome using RNA Sequel software [28]. Transcript levels were calculated as RPKM (Reads per kilobase cDNA per million fragments mapped). Differential expressed genes were analyzed using Cufflinks software (version 2.1.1) with fold change (cutoff = 2.0) [29], and considered statistically significant if the fold change was >2.0 and the FDR (False Discovery Rate) was <0.001.

### Real-time quantitative PCR analysis

Real-time qPCR was performed to confirm transcriptional levels of differently expressed genes obtained in the RNA-Seq analysis. Gene-specific primers were designed using primer3 online software Version.0.4.0 [30] and described in Table 1. The expression of the l-lactate dehydrogenase encoding gene (ldh) was measured using primers RA ldh-F/RA ldh-R (Table 1), and used as an internal control [31]. Total RNA was isolated from the WT strain CH3 and mutant strain RA1067 using Trizol reagent (Invitrogen, Carlsbad, CA, USA), according to the manufacturer’s instructions. All RNA samples were treated with TURBO DNA-free kit (Ambion, Grand Island, NY, USA) to remove DNA contamination. cDNA was synthesized using PrimeScript RT Master Mix (Takara). Real-time qPCR was carried out in Go Taq qPCR Master Mix (Promega, Fitchburg, WI, USA) using the following parameters: 95 °C for 2 min, 40 cycles of 95 °C for 15 s, 55 °C for 15 s and 68 °C for 20 s, followed by one cycle of 95 °C for 15 s, 60 °C for 15 s and 95 °C for 15 s. Reactions were performed in triplicate and run on the Mastercycler ep realplex4 apparatus (Eppendorf, Germany). Quantification of transcriptional level was calculated according to the 2^−ΔΔCt^ method.

### Statistical methods

Statistical analyses were performed using the GraphPad Prism, version 5.0 for Windows (GraphPad Software Inc., La Jolla, CA, USA). Adhesion and invasion assays, bacterial growth curves, bacterial loads in the blood of ducks, serum sensitivity assays, and RT-PCR were two tailed, and a *p* value of <0.05 was considered significant. Multi-group comparisons were carried out using ANOVA.

## Results

### Identification of the mutant strain RA1067

**Figure 1:**
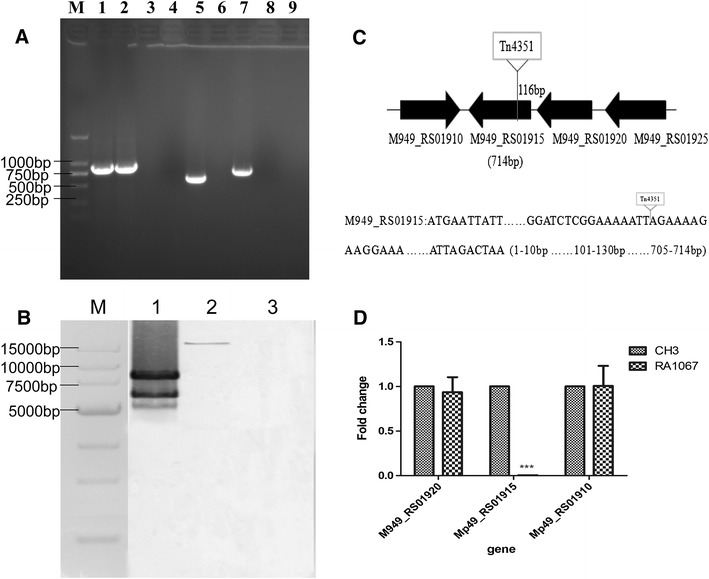
**Identification of the mutant strain RA1067. A** PCR amplification. M: Takara DL2000 marker; lanes 1–2: *R. anatipestifer* 16S rRNA was amplified from the WT strain CH3 (lane 1), the mutant strain RA1067 (lane 2), showing a 744-bp fragment of 16S rRNA; lanes 4–5: 644 bp fragment of the *erm* gene was amplified from the mutant strain RA1067 (lane 5), but not from the WT strain CH3 (lane 4); lanes 7–8: 714-bp fragment of M949_RS01915 gene was amplified from the WT strain CH3 (lane 7), but not from the mutant strain RA1067 (lane 8); lanes 3, 6 and 9 distilled water, as negative controls. **B** Southern blot analysis of the transposon Tn4351 insertion. Lane M Takara DL15000 marker; Lane 1, 10 μg of pEP4351 digested with XbaI (positive control). Lane 2, 10 μg of chromosomal DNA from mutant strain RA1067 digested with XbaI; Lane 3, 10 μg of chromosomal DNA from the WT strain CH3 digested with XbaI (negative control); each digested sample was resolved on a 0.7% agarose gel, and Southern blot was performed using a TnDIG labeled probe. **C** Schematic chart of Tn4351 insertion in the RA1067 chromosome. **D** Real time qPCR analysis. The changes of mRNA were expressed as fold expression and calculated using the comparative C_T_ (2^−∆∆CT^) method. Data were normalized to the housekeeping gene *ldh* and expressed as fold changes. Error bars represent standard deviations from three replicates (***, *p* < 0.001).

The mutant RA1067 that lacked reactivity with anti-LPS MAb was obtained by screening the transposon library using an indirect ELISA, and identified by PCR amplification using primers 16S rRNA F/16S rRNA R, Erm-F/Erm-R and RA1067-F/RA1067-R. As shown in Figure 1A, a 744-bp fragment of 16S rRNA was amplified from the WT strain CH3 (lane 1) and the mutant strain RA1067 (lane 2); a 714-bp fragment of *M949_RS01915* gene was amplified from the WT strain CH3 (lane 7), while no 714-bp fragments were amplified from the mutant strain RA1067 due to the transposon insertion (lane 8); a 644-bp fragment of the *erm* gene (Tn*4351* transposon contained the *erm* gene) was amplified from the mutant strain RA1067 (lane 5), but not from the WT strain CH3 (lane 4). Southern blot indicated that the mutant strain RA1067 contained a single Tn*4351* insertion in the chromosomal DNA (Figure 1B, lane 2). Sequencing analysis showed that the transposon was inserted at nucleotide position 116 bp of *M949_RS01915* gene, which consists of 714 nucleotides and encodes 237 amino acids (Figure 1C). Real-time qPCR analysis further confirmed *M949_RS01915* transcription was abolished in the mutant strain RA1067. Further investigation showed that the gene deletion had no effect on the transcription of chromosomally upstream *M949_RS01920* gene or downstream *M949_RS01910* gene (Figure 1D). BLAST analysis showed that the *M949_RS01915* gene was highly conserved in *R. anatipestifer*, which exhibits over 91% identity compared to other *R. anatipestifer strains.*

### Analysis of the bacterial LPS by silver staining and western blot

**Figure 2:**
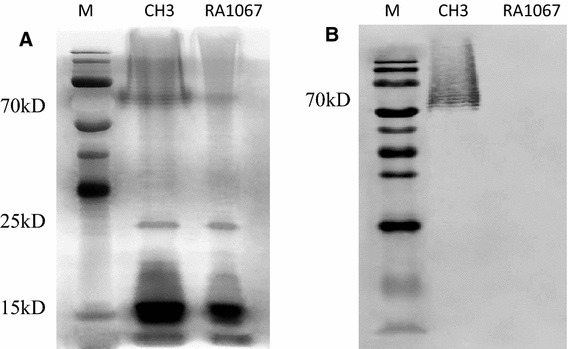
**Analysis of the bacterial LPS. A** Silver staining. **B** Western blot. Lane M: molecular weight marker; lane 1: LPS purified from the WT strain CH3; lane 2: LPS purified from the mutant strain RA1067.

LPS was purified from the WT strain CH3 and the mutant strain RA1067, and followed by silver staining and Western blot analyses. As shown in Figure 2A, LPS purified from the WT strain CH3 displayed a ladder-like pattern at 70 kDa, while it was absent in the mutant strain RA1067 LPS in the silver staining. No detectable protein bands were found in the gels by Coomassie blue staining (data not shown). Western blot analysis showed that a ladder-like pattern of O-antigen repeats to anti-LPS MAb was defected in the mutant strain RA1067 (Figure 2B). These results are beginning to suggest that the *R. anatipestifer* M949_RS01915 gene is involved in LPS O-antigen biosynthesis.

### Adhesion and invasion assays

**Figure 3:**
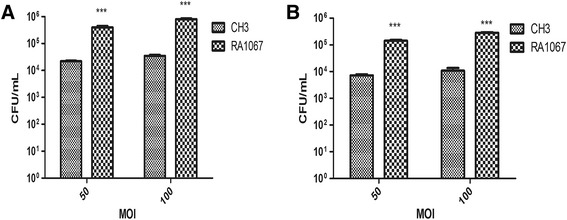
**Bacterial adherence and invasion assays.** Strains CH3 and RA1067 were tested on Vero cells. **A** Adherence assay; **B** invasion assay. The data represent the number of bacteria bound to or invaded into Vero cells in each well of a 24-well plate. The error bars represent mean ± standard deviations from three independent experiments (***, *p* < 0.001).

To determine whether disruption of *M949_RS01915* gene affects the bacterial adherence and invasion capacity, we compared bacterial adhesion and invasion abilities of the CH3 and RA1067 strains on Vero cells. The host cell-associated bacteria of the WT strain CH3 and mutant strain RA1067 were counted as 2.2 × 10^4^ CFU/well and 3.46 × 10^5^ CFU/well respectively when infected at 50 multiplicity of infection (MOI), and 3.49 × 10^4^ CFU/well and 8.05 × 10^5^ CFU/well respectively when infected at 100 MOI. The mutant strain RA1067 presented a 15.73- or 23.07-fold increased adherence ability in comparison with those of the WT strain CH3 (*p* < 0.001) (Figure 3A). After further 1-h incubation with 100 μg/mL gentamycin, invaded bacterial CFU of the mutant strain RA1067 were 1.41 × 10^5^ CFU/well at MOI of 50, and 2.87 × 10^5^ CFU/well at an MOI of 100, which were 19.58- and 26.57-fold higher than those of the WT strain CH3 (7.2 × 10^3^ CFU/well at an MOI of 50; 1.08 × 10^4^ CFU/well at an MOI of 100) (*p* < 0.001) (Figure 3B). The results showed that adhesion and invasion capacities of the mutant strain RA1067 were significantly increased in comparison with those of the WT strain CH3.

### Determination of bacterial growth curves and virulence

**Figure 4:**
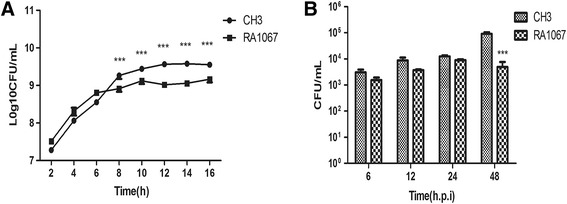
**Determination of the bacterial growth curves and virulence.** **A** Strains CH3 and RA1067 were grown in TSB at 37 °C with shaking respectively, and the bacteria numbers of CFU were measured at 2 h intervals. This experiment was repeated three times, and the data were presented as the means. Error bars represent standard deviations. Asterisks indicate statistically significant differences between groups. **B** Bacterial loads in blood of infected ducks with CH3 or RA1067 at 6, 12, 24 and 48 h post infection (hpi). After 72 hpi more than half of the WT CH3 infected ducks were dead, no data of bacterial loads in blood was collected. The error bars represent means ± standard deviations from six ducks. Asterisks indicate statistically significant differences between groups (***, *p* < 0.001).

The growth of the mutant strain RA1067 was significantly decreased at the late stage of the growth when compared to those of the WT strain CH3. The mutant strain RA1067 reached the plateau of 1.32 × 10^9^ CFU/mL at 8-h growing in TSB, while the WT strain CH3 reached the plateau of 3.68 × 10^9^ CFU/mL at 12-h growing in TSB. The bacterial numbers of the mutant strain RA1067 at the plateau was about 2.79-fold decreased in comparison with that of the WT strain CH3 (Figure 4A). Bacterial virulence was evaluated based on bacterial LD_50_ determination using 17-day-old Cherry Valley ducks. The LD_50_ for the mutant strain RA1067 was 2.74 × 10^10^ CFU, which was more than 365 times attenuated virulence than that of the WT strain CH3 (7.50 × 10^7^ CFU). In addition, the bacterial loading experiment further confirmed the attenuated virulence of the mutant strain RA1067. As shown in Figure 4B, the bacterial loads in the blood of ducks infected with the mutant strain RA1067 were decreased significantly in comparison with those of the ducks infected with the WT strain CH3 at 48 h post infection (hpi) (*p* < 0.001).

### The mutant strain RA1067 displayed a higher sensitivity to normal duck sera

**Figure 5:**
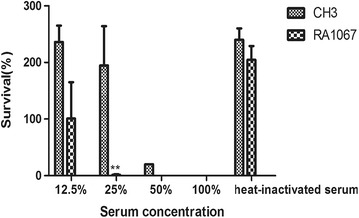
**Bacterial serum resistance assay.** Bacteria were incubated with the normal duck sera at different dilutions at 37 °C, and were enumerated at 30 min incubation. A significant reduced resistance of RA1067 to the normal duck sera than that of CH3 was shown (**, *p* < 0.01). The survival rate (%) was calculated as follows: (bacterial CFU with sera treatment/bacterial CFU with PBS treatment) × 100.

To determine whether the *M949_RS01915* gene is involved in serum resistance of the WT strain CH3, we compared the WT strain CH3 and mutant strain RA1067 for their abilities to resist the complement-mediated killing. The results showed that 25% diluted serum was effective in killing the mutant strain RA1067, but not the WT strain CH3, indicating that the mutant strain RA1067 was more sensitive to normal duck sera than that of the WT strain CH3 (Figure 5).

### Cross-protection experiment

To investigate the cross-protection against *R. anatipestifer* serotypes 1, 2 and 10 strains, the RA 1067 vaccinated ducks were challenged with virulent *R. anatipestifer* strains WJ4 (serotype 1), Yb2 (serotype 2) and HXb2 (serotype 10) at 10 LD_50_ respectively. When challenged with strains WJ4, Yb2, or HXb2 at day 14 post vaccination, the ducks in group 1 were 7/8, 6/8, or 7/8 protected respectively; the ducks in group 2 were 7/8, 2/8, or 5/8 protected respectively. The protection rate of the WT strain CH3 vaccine against WJ4 challenge was the same as that of the mutant strain, however, the protection against Yb2 and HXb2 challenges was 50 and 25% lower than those of the mutant strain RA 1067 vaccine respectively. The ducks in group 3 (control group) were all dead within 7 days post challenge (Table 2). These results indicate that RA1067 provided cross-protection against challenge with strains from *R. anatipestifer* serotypes 1, 2 and 10, further confirmed its altered antigenicity.

**Table 2:** **Cross-protection experiment**

Groups	Challenge strains	Total no. of ducks	Protection rate (protected no./total no.)^a^
1 (RA1067 vaccine)	WJ4	8	7/8
	Yb2	8	6/8
	HXb2	8	7/8
2 (CH3 vaccine)	WJ4	8	7/8
	Yb2	8	2/8
	HXb2	8	5/8
3 (Saline in adjuvant)	WJ4	8	0
	Yb2	8	0
	HXb2	8	0

^a^ Challenge dose was 10 LD_50_ for each strain.

### Determination of the differentially expressed genes

Strand-specific Illumina RNA-Seq analysis was used to investigate the differentially expressed genes between the WT strain CH3 and mutant strain RA1067. In total, 9 genes were up-regulated and 10 genes were down-regulated in the mutant strain RA1067 in comparison to the WT strain CH3 (Table 3), which were differentially expressed by >fivefold based on RNA-Seq analysis. Real-time qPCR further confirmed *M949_RS01940*, *M949_RS09405* genes were up-regulated over twofold at transcriptional levels. The proteins encoded by *M949_RS01940* and *M949_RS09405* genes were annotated as hypothetical protein and transcriptional regulator respectively. Three genes of *M949_RS07580*, *M949_RS10455* and *M949_RS00790* were further verified to be down-regulated over twofold at transcriptional levels. The coding products were annotated as polysaccharide biosynthesis protein CapD, DNA-binding protein and galactose-binding protein respectively. The polysaccharide biosynthesis protein CapD is responsible for bacterial capsule polysaccharide biosynthesis. The results indicate that the *M949_RS01915* gene may regulate the genes mainly responsible for bacterial polysaccharide biosynthesis.

**Table 3:** **Real-time qPCR verification of differentially expressed genes in mutant strain RA1067**

Gene locus^a^	Description of genes	2^−ΔΔCt^
M949_RS01940	Hypothetical protein	3.71
M949_RS09405	Transcriptional regulator	3.09
M949_RS10515	Hypothetical protein	1.92
M949_RS00810	Hypothetical protein	1.88
M949_RS04680	TonB-dependent receptor	1.46
M949_RS03025	TonB-denpendent receptor	1.03
M949_RS10460	Hypothetical protein	0.99
M949_RS09795	Hypothetical protein	0.88
M949_RS00955	Hypothetical protein	0.84
M949_RS00980	Hypothetical protein	0.72
M949_RS05615	DNA-binding protein	0.71
M949_RS05620	Hypothetical protein	0.68
M949_RS00870	Head morphogenesis protein	0.67
M949_RS07550	thij/pfpi domain-containing protein	0.61
M949_RS10465	Hypothetical protein	0.49
M949_RS10510	S41 family peptidase	0.44
M949_RS10455	DNA-binding protein	0.31
M949_RS00790	Galactose-binding protein	0.23
M949_RS07580	Polysaccharide biosynthesis protein CapD	0.02

^a^ Based on *R. anatipestifer* CH3 genome (accession number: CP006649).

## Discussion

DNA transposition was used as a powerful approach for the generation of appropriate knockout mutations for functional gene analysis. In a previous study, random transposon mutagenesis was used to successfully identify 33 genes involved in biofilm biosynthesis [32]. In the present report, the mutant strain RA1067 was identified by screening the library of random Tn*4351* transposon mutants using anti-LPS MAb. The mutant lacks reactivity with anti-LPS MAb in an indirect ELISA. Further investigations revealed that the mutant showed different growth characteristics in TSB, increased capacity of adhesion and invasion, increased sensitivity to normal duck serum, and significantly attenuated virulence in ducks. Western blot indicated a distinct loss of ladder-like pattern in RA1067 mutant LPS, compared to those in CH3 LPS. BLAST searches showed that the *M949_RS01915* gene is highly conserved among *R. anatipestifer* strains.

LPS isolated from the WT strain CH3 and mutant strain RA1067 were analyzed by SDS-PAGE followed by silver staining and Western blot. Silver staining showed that the RA1067 LPS lacked the ladder-like patterns as presented in the WT strain CH3 LPS. In addition, the destructed LPS lost the binding activity with anti-LPS MAb. The results above suggest that *M949_RS01915* takes part in biosynthesis of integrated O-antigen in *R. anatipestifer*. Consequently, we investigated the adherence and invasion capability of the RA1067 on Vero cells as described previously. The result manifested that the adhesion and invasion capacities of the RA1067 were sharply increased compared to those of the WT strain CH3. Moreover, the virulence of the RA1067 was more than 365 times attenuated than that of the WT strain CH3 based on the LD_50_ determination and the bacterial loads in blood of ducks infected with RA1067 were significantly decreased at 48 h, compared with those of ducks infected with CH3. Therefore, we demonstrate that the pathogenicity of the RA1067 was decreased compared with that of the WT strain CH3. These findings were in agreement with previous studies describing that O-antigen of LPS is essential for bacterial adherence and virulence for *Salmonella* [33]. Bacterial adhesion and invasion abilities of the mutant strain RA1067 were significantly enhanced, compared to those of the WT strain. In addition, the LPS pattern was altered, suggesting that the RA1067 may take a different entry route to adhere and invade cells as previously reported [34]. O-antigen also plays an essential role in protecting bacteria from serum complement-mediated killing [35, 36]. In this study, inactivation of the *M949_RS01915* gene resulted in significantly increased sensitivity to normal duck serum killing, while the WT strain CH3 presented a dose-dependent resistance to the normal duck serum killing. This result has also been shown previously, indicating that the deficiency of LPS O-antigen chains of *R. anatipestifer* exhibit more sensitivity to normal duck serum killing [37].

As we all know, there is no effective cross-protection among different serotypes of *R. anatipestifer*. Our results show that the inactivated RA1076 vaccine protected the ducks from challenge with *R. anatipestifer* strains WJ4 (serotype 1), Yb2 (serotype 2) and HXb2 (serotype 10) at 7/8, 6/8 and 7/8 respectively; 50 and 25% were improved compared with inactivated CH3 vaccine challenged with *R. anatipestifer* strains Yb2 (serotype 2) and HXb2 (serotype 10). It has been noted that O-antigen, one of the most variable cell constituents, is a serotype feature of Gram-negative bacteria and defines their O-serospecificity [38]. This study demonstrates that the inactivated vaccine provides effective cross-protection because of deficiency in O-antigen of the mutant strain RA1067, further confirming that the antigenicity of the RA1067 was altered.

RNA-Seq technology was applied to analyze the differential gene expressions in the mutant strain RA1067. In our RNA-Seq analysis, we found that deletion of *R. anatipestifer* CH3 *M949_RS01915* gene resulted in up-regulation of 9 genes and down-regulation of 10 genes, respectively. Real time qPCR verification further confirmed that two genes were up-regulated and three genes were down-regulated by over twofold. Protein encoded by up-regulated *M949_RS09405* is BlaI family transcriptional regulator, penicillinase repressor. As for BlaI family transcriptional regulator in *Staphylococcus aureus*, *blaI* is predicted to encode a repressor protein and regulate the production of both PBP 2a and 1-lactamase [39]. Protein encoded by up-regulated *M949_RS01940* gene belongs to the PhnB protein family. Many proteins of the PhnB protein family have been annotated as gene 3-demethylubiquinone-9 3-methyltransferase enzymes, which is necessary for the use of phosphonates (Pn) supporting bacterial growth on alkylphosphonates as a sole phosphorus source in *Escherichia coli* [40]. The product of down-regulated *M949_RS10455* gene is DNA-binding protein. It has been reported that some members of DNA-binding proteins are involved in DNA binding, supercoiling and DNA compaction. In addition to architectural roles, some DNA-binding proteins also play regulatory roles in DNA replication and repair, and act as global transcriptional regulators in many bacteria [41]. Galactose binding protein encoded by the down-regulated *M949_RS00790* gene belongs to a group of soluble proteins that can be found in the periplasmic space of Gram-negative bacteria; some members of these proteins are responsible for stimulating chemotaxis in low nutrient environments and participate in active transport of small molecules and ions from the periplasm to the cytoplasm [42]. Down-regulated gene *M949_RS07580* was annotated to encode polysaccharide biosynthesis protein CapD, which is involved in “metabolic process, DNA binding, catalytic activity”. Polysaccharide biosynthesis protein CapD, in *Enterococcus faecium*, was identified as a virulence factor and involved in bacterial growth, biosynthesis of surface polysaccharides [43]. It has also been reported that polysaccharide biosynthesis protein CapD is associated with the Gram-negative bacterial capsule, high-molecular-weight capsular polysaccharides are critical for bacterial resisting against opsonophagocytosis and complement-mediated killing [44, 45]. The CapD protein is required for biosynthesis of type 1 capsular polysaccharide in *Staphylococcus* spp, and Group 1 and 4 capsules are related to LPS O-antigens [45–47]. Based on these results, for the mutant strain RA1067, we suggest that disruption of the *M949_RS01915* gene mainly leads to down-regulation of the *M949_RS07580* gene, which results in reduced formation of type 1 capsular polysaccharide and LPS O-antigens. However, the mechanism of *M949_RS01915* gene regulation of *M949_RS07580* gene is currently unknown and needs further investigation.

Bacterial LPS is biosynthesized by several dozens of genes [15, 47]. We previously reported three genes are associated with the *R. anatipestifer* LPS biosynthesis [16–18]. In this study, one more LPS biosynthesis associated gene *M949_RS01915* was identified. We used the anti-LPS monoclonal antibody to screen the LPS mutant defective in the LPS structure to identify the LPS biosynthesis associated genes, which will benefit for the future LPS structure analysis, as well as vaccine and pathogenesis studies. In conclusion, we identified a transposon mutant RA1067 with deletion of the *M949_RS01915* gene. We demonstrate that the *M949_RS01915* gene is involved in LPS O-antigen synthesis, bacterial virulence and gene regulation in *R. anatipestifer.* Further experiments will be needed to characterize the *M949_RS01915* gene to clarify its functions and the exact mechanism in bacterial virulence and gene regulation.
